# Neuroprotective Role of the Ron Receptor Tyrosine Kinase Underlying Central Nervous System Inflammation in Health and Disease

**DOI:** 10.3389/fimmu.2018.00513

**Published:** 2018-03-19

**Authors:** Adwitia Dey, Joselyn N. Allen, James W. Fraser, Lindsay M. Snyder, Yuan Tian, Limin Zhang, Robert F. Paulson, Andrew Patterson, Margherita T. Cantorna, Pamela A. Hankey-Giblin

**Affiliations:** ^1^Center for Molecular Immunology and Infectious Diseases, Department of Veterinary and Biomedical Sciences, The Pennsylvania State University, University Park, PA, United States; ^2^CAS Key Laboratory of Magnetic Resonance in Biological Systems, State Key Laboratory of Magnetic Resonance and Atomic and Molecular Physics, National Centre for Magnetic Resonance in Wuhan, Wuhan Institute of Physics and Mathematics, University of Chinese Academy of Sciences, Wuhan, China

**Keywords:** neuroinflammation, tyrosine kinase, macrophage, experimental autoimmune encephalomyelitis, diet-induced obesity, central nervous system

## Abstract

Neurodegeneration is a critical problem in aging populations and is characterized by severe central nervous system (CNS) inflammation. Macrophages closely regulate inflammation in the CNS and periphery by taking on different activation states. The source of inflammation in many neurodegenerative diseases has been preliminarily linked to a decrease in the CNS M2 macrophage population and a subsequent increase in M1-mediated neuroinflammation. The Recepteur D’Origine Nantais (Ron) is a receptor tyrosine kinase expressed on tissue-resident macrophages including microglia. Activation of Ron by its ligand, macrophage-stimulating protein, attenuates obesity-mediated inflammation in the periphery. An *in vivo* deletion of the ligand binding domain of Ron (Ron^−/−^) promotes inflammatory (M1) and limits a reparative (M2) macrophage activation. However, whether or not this response influences CNS inflammation has not been determined. In this study, we demonstrate that in homeostasis Ron^−/−^ mice developed an inflammatory CNS niche with increased tissue expression of M1-associated markers when compared to age-matched wild-type (WT) mice. Baseline metabolic analysis of CNS tissue indicates exacerbated levels of metabolic stress in Ron^−/−^ CNS. In a disease model of multiple sclerosis, experimental autoimmune encephalomyelitis, Ron^−/−^ mice exhibit higher disease severity when compared to WT mice associated with increased CNS tissue inflammation. In a model of diet-induced obesity (DIO), Ron^−/−^ mice exhibit exacerbated CNS inflammation with decreased expression of the M2 marker Arginase-1 (Arg-1) and a robust increase in M1 markers compared to WT mice following 27 weeks of DIO. Collectively, these results illustrate that activation of Ron in the CNS could be a potential therapeutic approach to treating various grades of CNS inflammation underlying neurodegeneration.

## Introduction

Neuroinflammation is a critical underlying component of neurodegenerative diseases and exacerbates disease symptoms associated with Alzheimer’s (AD), Parkinson’s (PD), multiple sclerosis (MS), and with homeostatic aging (1, 2). Neuroinflammation is a biological response to physiological perturbations whereby the central nervous system (CNS) coordinates a series of immunological responses in order to return to a homeostatic state. Neuroinflammation can be acute, such as in ischemic stroke, or chronic which supports long-term neuronal loss exhibited in progressive autoimmune diseases such as MS or neurogenerative states such as AD (3). In degenerative diseases, these processes persist, leading to uncontrolled inflammation that becomes toxic to the CNS tissue proper, propels breakdown of the blood–brain barrier (BBB) and promotes subsequent tissue injury (4). We now know that metabolic syndrome and obesity are potent stimuli for the earlier onset of neurodegeneration in AD etiology (5). A longitudinal study of adults illustrated that individuals who exhibit higher BMI in their 30s and 40s have an earlier onset of dementia associated with Alzheimer’s (5). In all cases of neuroinflammation, CNS macrophages such as tissue resident microglia are key innate immune cells that closely regulate the inflammatory process (6, 7).

Macrophages take on various activation states to carry out their respective functions in health and disease (8–12). Tissue-resident macrophages exhibit an alternatively activated M2 phenotype which promotes tissue regeneration, wound healing, and repair (13, 14). The classically activated M1 macrophage is induced by various environmental stimuli including the engagement of pattern recognition receptors by pathogen-associated molecular patterns, such as lipopolysaccharide (LPS) (15). CNS macrophages or microglia are primarily yolk-sac derived and are maintained in the CNS proper throughout adulthood. Microglia do not require the transcription factor Myb for development, indicating these macrophage populations develop independent from contribution by bone marrow progenitors (16, 17). In the steady state, microglia are in a quiescent M2 or downregulated phenotypic state important for housekeeping functions such as tissue surveillance (18). *In vitro* classical M1 or alternative M2 macrophage activation can be induced in microglia by LPS and/or interferon gamma (IFNγ) and IL-4, respectively (19). However, whether this phenotypic switching of resident microglia to M1 or M2 occurs *in vivo* remains controversial.

Studies investigating progressive neurodegeneration in AD, PD, and even aging-associated inflammation, have demonstrated a loss of the M2 phenotype underlying the neuroinflammation in these disease states (20). Studies in a murine spinal cord injury (SCI) model and a murine model of MS, experimental autoimmune encephalomyelitis (EAE), highlight the importance of the engagement of both M1 and M2 phenotypes, postulated to act in synergy in order to mitigate and resolve the onset of inflammation. Contrary to existing dogma, recent studies of aggressive autoimmune diseases, severe CNS amyloidosis, and aggressive neuronal injury have demonstrated an essential role for myeloid cell trafficking to aid in phagocytic activity and appropriate clearance of debris (21, 22). Nonetheless, in chronic neuroinflammation, the classically activated M1 phenotype counterbalances the M2 phenotype and disrupts the normal inflammatory CNS milieu, thus supporting uncontrolled inflammation and perpetuation of the disease state.

During progressive neuroinflammation pro-inflammatory cytokines engage matrix metalloproteinases (MMPs), particularly MMP9 and to a lesser extent MMP2, which stimulate the degradation of the CNS proper (23, 24). With prolonged inflammation, MMPs degrade basal lamina components including heparan sulfate, laminin, fibronectin, and type IV collagen (23, 25). Without an intact basal lamina, the CNS is vulnerable to trafficking of foreign substances into the tissue proper. In cases of persistent or chronic inflammation, there is a breakdown of the immunoprivileged BBB facilitated in part by MMPs, which enables immune cell trafficking (26). Predominant populations of trafficked monocytes exhibit the M1 phenotype and in chronic cases, exacerbate the existing tissue inflammation. Altogether, the heterogenic balance of macrophages is key to regulating neuroinflammation.

Ron is a growth factor receptor of the Met protooncogene family of transmembrane tyrosine kinases, expressed on tissue-resident macrophages including CNS microglia (27). Macrophage-stimulating protein (MSP), produced by the liver in its inactive form, is a hepatocyte-like growth factor that is the ligand for Ron (28). The activation of MSP is carried out by proteolytic processing at sites of inflammation. During an inflammatory cascade, the MSP-dependent activation of Ron on tissue resident macrophages has been shown to support anti-inflammatory processes and subsequent tissue repair. Previous studies in our lab have demonstrated that an *in vivo* deletion of the extracellular ligand binding domain of the Ron receptor (Ron^−/−^) promotes inflammatory (M1) and limits reparative (M2) macrophage activation (29–32). We have further shown that MSP-dependent activation of Ron attenuates obesity-induced chronic inflammation (27). A previous study by Stella et al. demonstrated that *in vivo* administration of exogenous MSP or the transplantation of cells producing MSP at the proximal end of an injured “resected” neuron attenuates neuronal atrophy following axotomy induced injury (33). Exogenous MSP is a neurotrophic factor which promotes the growth and survival of peripheral sympathetic neurons at various stages of development (34). However, little is known about the potential function of Ron in CNS inflammation.

In the studies described here, we demonstrate that Ron is expressed on microglia and that loss of Ron responsiveness to MSP induces neuroinflammation in the CNS. Ron expression in the CNS supports metabolic homeostasis, suggesting that Ron is also important for metabolic regulation in the CNS. A loss of Ron exacerbates disease-mediated neuroinflammation in a model of MS and accelerates the degree of inflammation in a chronic inflammatory model of diet-induced obesity (DIO). These results demonstrate that Ron plays a key protective role in CNS health and homeostasis.

## Materials and Methods

### *In Vitro* Stimulation Assays

CHME-3 cells (human fetal microglia cell line) were obtained from Dr. A. Henderson (Boston University). Recombinant human MSP (Cys672Ala) protein was obtained from R&D Systems (Minneapolis, MN, USA), and culture grade LPS was obtained from Sigma-Aldrich (St. Louis, MO, USA). Cells were incubated with MSP (100 ng/mL) overnight followed by 4-h stimulation with LPS (100 ng/mL). In the EAE model, cells were restimulated *in vitro* with myelin oligodendrocyte glycoprotein (MOG). At day 14, inguinal lymph nodes (LNs) and spleens were collected and a single-cell suspension prepared. Splenocytes and LN cells (2 × 10^6^ cells mL^−1^) were cultured for 72 h with MOG peptide (20 µg mL^−1^), cells were collected, centrifuged, and suspended in TRIzol reagent for RNA extraction.

### Mouse Models

#### Mice

All mice are on a C57BL/6 background. Wild-type (WT) mice were purchased from Jackson Laboratories (Bar Harbor, ME, USA). Both male and female mice were used for DIO models while females were used for the murine model of MS. Mice with a targeted disruption of the Ron gene (Ron^−/−^), whereby the ligand binding domain of Ron is deleted, were generated as previously described (27). For homeostasis/baseline study, WT and Ron^−/−^ mice were placed lab-diet standard chow provided by Penn State Animal Care Facilities. These experimental protocols were approved by the Institutional Animal Care and Use Committee (IACUC) at The Pennsylvania State University. Mice were euthanized by CO_2_ narcosis. Following euthanasia, end point blood was collected for circulating immune cells, and tissues were harvested for analysis.

#### Experimental Autoimmune Encephalomyelitis

Experimental autoimmune encephalomyelitis is a widely used experimental mouse model of MS as previously described (35). For EAE induction, 8- to 10-week-old WT and Ron^−/−^ mice were subcutaneously injected with 200 µg MOG 35–55 (amino acid sequence, MEVGWYRSPFSRVVHLYRNGK; Anaspec, Fremont, CA, USA) emulsified in Freund’s adjuvant (Difco, Detroit, MI, USA) supplemented with attenuated *Mycobacterium tuberculosis* H37RA (Difco) to 4 mg mL^−1^. At 0 and 48 h post immunization, the mice were injected intraperitoneally with 200 ng pertussis toxin (List Biological Laboratories; Campbell, CA, USA) in 100 µL PBS. The clinical symptoms of EAE were evaluated daily and scored as follows: 0, no clinical signs; 1, loss of tail tonicity; 2, partial hind limb paralysis; 3, total hind limb paralysis; 4, hind and some forelimb paralysis; 5, moribund or dead (11). Mice with EAE score of 4 were sacrificed according to IACUC guidelines. Following EAE induction, animals are assessed at least twice daily for clinical symptoms and tissues collected following 14 (peak disease state in WT) or 28 days post EAE induction.

#### Diet-Induced Obesity

Wild-type and age-matched Ron^−/−^ mice were fed custom ordered HFD (Diet No. F3282, Bioserv) for the indicated number of weeks (18 or 27). Nutritional breakdown of HFD consisted of 36% fat from lard, 35.7% carbohydrate, and 20.5% protein. All animals were maintained in a humidity and temperature-controlled room on a 12-h light–dark cycle. Circulating blood was analyzed by a Hemavet (Drew Inc.).

### In Study—Metabolic Analysis

Fasting glucose levels were measured on chow and HFD fed mice, that were deprived of food (morning fast) for 5–6 h. For the glucose tolerance test, glucose (2 g/kg) was administered intraperitoneally. Tail blood was collected at the indicated time points, and blood glucose concentration was determined on the OneTouch Ultra blood glucose monitoring system.

### RNA Isolation and Quantitative Real-Time (RT) PCR Analysis

For RNA analysis in all models, messenger RNA was extracted from whole tissues such as brain, spinal cords, single-cell suspension pellets of LN and spleen, and other mentioned cell types using TRIzol Reagent (Invitrogen) as instructed by the manufacturer. RNA was quantified by A260 absorbance. For cDNA synthesis, 1.5–2 µg of RNA was reverse transcribed using the High Capacity Reverse Transcription Kit (Applied Biosystems, Foster City, CA, USA). RT-PCR was performed on the resultant cDNA using FAM labeled TaqMan probes (Applied Biosystems, Foster City, CA, USA) and the 7900HT Fast Real-Time PCR System (Applied Biosystems, Foster City, CA, USA). All TaqMan probes used for RT PCR were purchased from Applied Biosystems (Foster City, CA, USA). Results were expressed as CT values normalized to GAPDH.

### Flow Cytometry and Analysis

Central nervous system tissues including brain and spinal cord were collected and macerated with cold PBS in small petri dishes that were kept cold (on ice). Cell suspensions were strained with a 70-µm strainer and mononuclear cells are isolated with 30% percoll gradient. The pelleted cells were washed and pre-blocked for Fc receptors using anti-CD16/32 (Fc-block). Cells were then incubated with monoclonal antibody at 4°C for 30 min, after which cells were washed with PBS containing 2% FBS. For flow cytometry antibodies the CD16/32 Fc-block, PE conjugated anti-F4/80, Pacific Blue conjugated anti-CD45, APC-Cy7 conjugated anti-Ly6C and/or Alexa 488 conjugated anti-CD206 were all obtained from Biolegend (San Diego, CA, USA). Anti-mouse Ron was purchased from R&D Systems (Minneapolis, MN, USA), and the secondary antibody was Alexa 488 conjugated to anti-goat for Ron. Stained cells were analyzed on a BD LSR Fortessa (BD Biosciences), and the flow output was analyzed with FlowJo software (Tree Star).

### NMR Spectroscopy and Analysis

Central nervous system tissues from WT and Ron^−/−^ were weighed and prepared as noted in previous experiments (27, 36). Approximately 200 µL of the prepared CNS lysate was mixed with 400 µL of saline solution containing 30% DO and vortexed, centrifuged (11,180 *g*, 10 min, 4°C), and 550 µL of the sample was transferred into 5 mm NMR tubes. ^1^H NMR spectra of the prepared CNS samples was acquired and spectral data processing and multivariate analysis was performed as previously described (36). The WT to Ron^−/−^ metabolites ratio was calculated by using values derived from normalizing the NMR peak area normalized to total integration for each sample.

### MOG-Induced Cytokine Assessment With ELISA

Inguinal LNs and spleens were collected and a single-cell suspension was prepared. Splenocytes and LN cells (2 × 10^6^ cells mL^−1^) were cultured for 72 h with MOG peptide (20 µg mL^−1^). To detect IFN-γ and IL-10 in culture supernatants, OptEIA kits were used (BD Pharmingen, San Jose, CA, USA). Detection of IL-17 by ELISA was performed using anti-mouse IL-17 and biotin-anti-mouse IL-17 from BD Pharmingen. The limits of detection for all cytokines were 31 pg mL^−1^.

### Statistics

Values are expressed as mean ± SEM. Statistical analysis was performed using unpaired Student’s *t*-test, paired Student’s *t*-test, and one-way or two-way ANOVA. Differences were considered significant at *P* < 0.05 (*P < 0.05, **P < 0.01, ***P < 0.001). All analysis was performed using GraphPad Prism 5.0 (San Diego, CA, USA).

## Results

### Ron Is Expressed on Microglia and Promotes Homeostasis in the CNS

**Figure 1:**
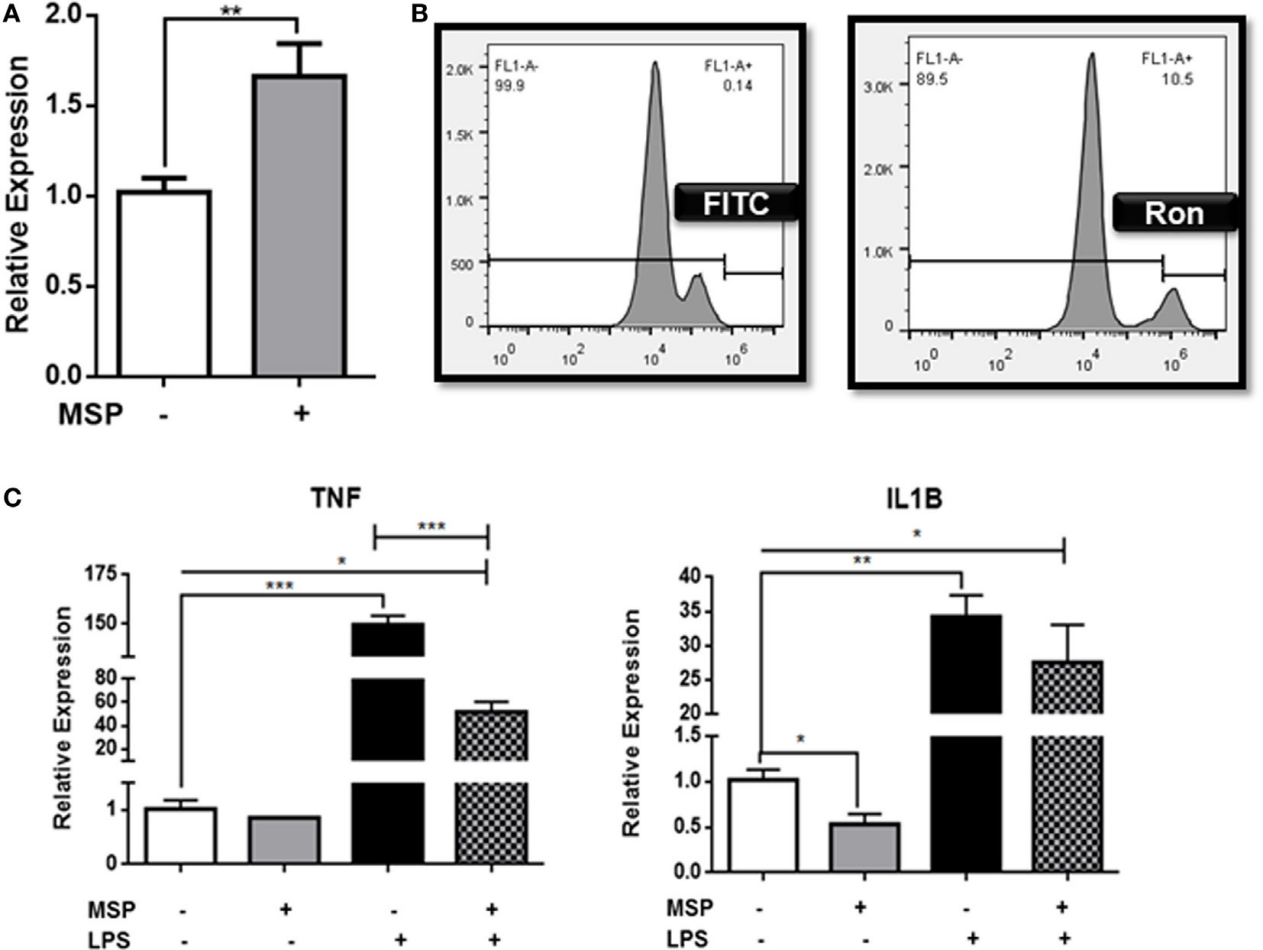
Ron suppresses lipopolysaccharide (LPS)-induced upregulation of TNFα in CHME-3 cells. **(A)** CHME-3 cells were cultured in the presence or absence of macrophage-stimulating protein (MSP) and expression of Ron was assessed by qPCR. **(B)** Cell surface expression of Ron on CHME-3 cells. **(C)** CHME-3 cells were stimulated for 4 h with LPS in the presence or absence of MSP. TNFα and IL-1B expression was assessed by qPCR. Data are representative of three individual experiments (*P < 0.05, **P < 0.01, ***P < 0.001).

Ron is widely expressed in the brain and spinal cord on both microglia and neurons (37). In order to determine the function of Ron in microglia, CHME-3 cells were pretreated with the Ron agonist MSP, and the expression of Ron mRNA was assessed by qPCR (Figure 1A). Our results confirm expression of Ron in microglial cells and demonstrate that MSP stimulation induces expression of Ron in a positive feedback manner. Experimental analysis with flow cytometry confirmed the cell surface expression of Ron on human microglia (Figure 1B). In order to determine whether Ron attenuates M1-mediated inflammation in microglia, CHME-3 cells were treated with LPS for 4 h in the presence or absence of MSP. LPS-induced expression of mRNA for both TNFα and IL-1B in the CHME-3 cells and MSP inhibited the LPS-induced TNFα and IL-1B expression significantly (Figure 1C). Here, we confirmed the expression of Ron on CNS microglia and MSP-dependent activation of Ron is able to attenuate microglial inflammation.

**Figure 2:**
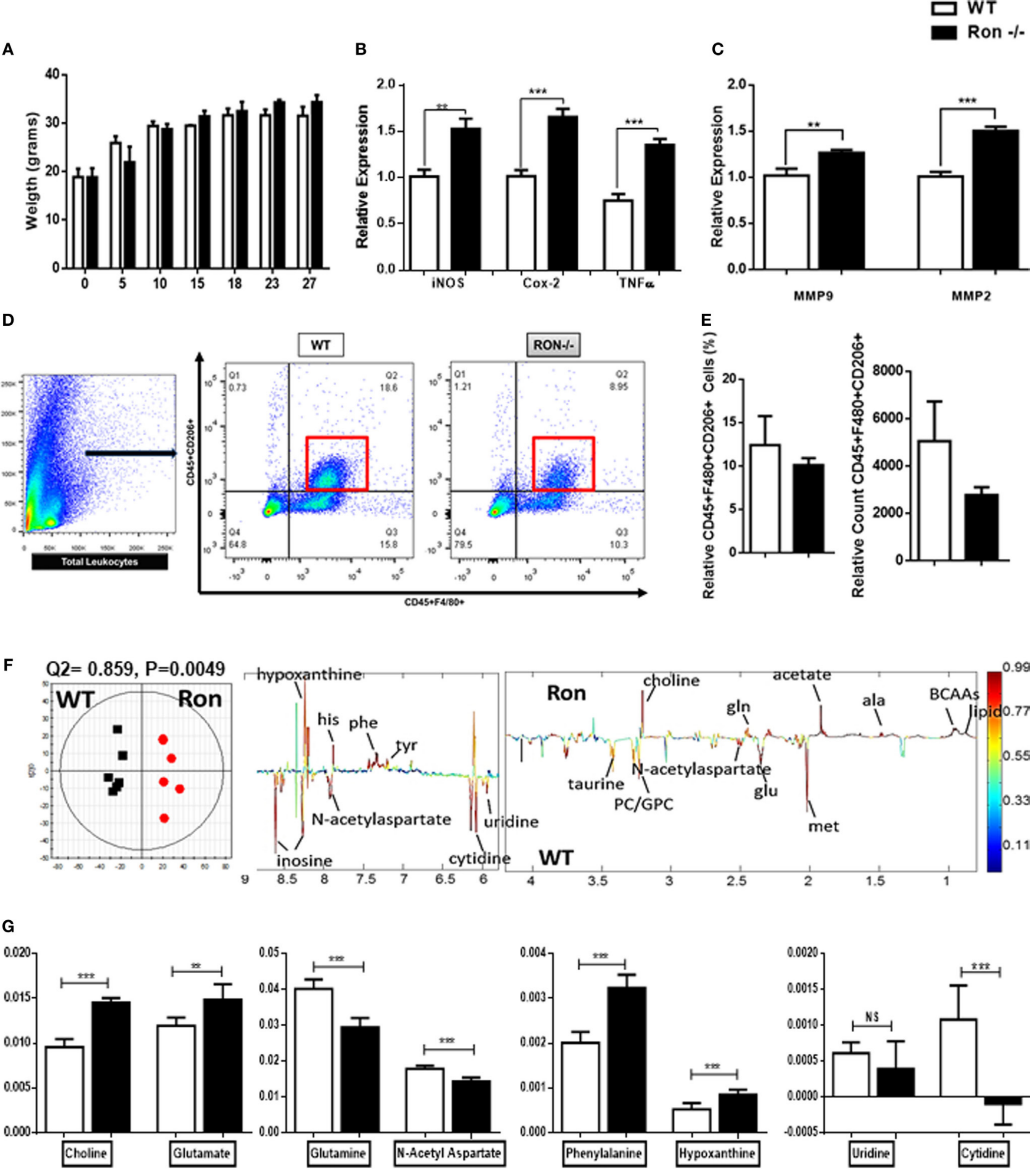
Ron promotes homeostasis in the central nervous system (CNS). **(A)** Weight of wild-type (WT) and Ron^−/−^ mice at 27 weeks of age (*n* = 16 mice/genotype). **(B,C)** Expression of pro-inflammatory genes and MMPs in the CNS of WT and Ron^−/−^ at 27 weeks of age (*n* = 6–8 mice/genotype). **(D,E)** Immune cells were isolated from the CNS of WT and Ron^−/−^ mice at 27 weeks and the presence of CD45^+^F480^+^CD206^+^ macrophages was assessed by flow cytometry (*n* = 4 mice/genotype). **(F)** OPLS-DA scores plot (left) and coefficient plot (right) derived from ^1^H NMR spectra of CNS samples from WT and Ron^−/−^ mice at 27 weeks of age. **(G)** Relative abundance of CNS metabolites measured by ^1^H NMR data from WT and Ron^−/−^ mice at 27 weeks of age (*n* = 6 mice/genotype) Abbreviations: BCAAs, branched chain amino acids; ala, alanine; met, methionine; glu, glutamate; gln, glutamine; tyr, tyrosine; phe, phenylalanine; his, histidine; MMPs, matrix metalloproteinases (*P < 0.05, **P < 0.01, ***P < 0.001).

In order to examine the function of Ron in the CNS, WT, and Ron^−/−^ mice were examined at various weeks of age up to 27 weeks. There was no significant difference in weight between Ron^−/−^ and WT mice (Figure 2A) through 27 weeks of age. CNS tissues (brain proper) were collected from Ron^−/−^ and WT mice at 27 weeks of age and analyzed for macrophage-related gene expression. Ron^−/−^ CNS exhibited M1-mediated inflammation, associated with increased expression of iNOS, COX-2, and TNFα (Figure 2B). Focal CNS inflammation enhances tissue degradation by MMPs at the CNS parenchymal border (26). Inflammatory cytokines TNFα and IL-1B induce focal MMP activation and production by activated glial tissue such as astrocytes (26). Consistent with the increase in TNFα expression, we observed increased expression of MMP2 and MMP9 in CNS tissue from Ron^−/−^ mice, indicative of tissue degradation in the CNS (Figure 2C). In order to determine whether there was a loss of M2 macrophages in the CNS of Ron^−/−^ mice, we examined the cell surface expression of CD206, a marker of M2 macrophage activation on CD45^+^F4/8^+^ macrophages in brains from 27-week-old WT and Ron^−/−^ mice. We observed a trend toward decreased M2 macrophage populations in Ron^−/−^ brains compared to the brains from WT age-matched mice (Figures 2D,E).

Metabolic profiling of the cerebrospinal fluid and plasma has been integral for fingerprinting early progression of pathogenesis in neurodegenerative diseases including Alzheimer’s (38–40). In humans, metabolite analysis are a non-invasive source for studying underlying mechanisms of neuroinflammation. The CNS is a very metabolic organ and shifts in metabolites are key indicators of neuronal and tissue injury (41). Orthogonal projection to latent structure-discriminant analysis (OPLS-DA) was done comparing the ^1^H-NMR data of CNS tissue lysates collected from 27-week-old WT and Ron^−/−^ mice (Figure 2F). The model quality indicators showed that the metabolites in the CNS were distinctive among the two genotypes (Figure 2F). Metabolic indicators of CNS stress such as choline, glutamate, phenylalanine, and hypoxanthine were increased in the Ron^−/−^ tissues whereas indicators of neuronal health such as glutamine and *N*-acetyl aspartate were decreased (Figure 2G) (42–44). Despite enhanced tissue inflammation in CNS tissue from Ron^−/−^ mice, metabolic indicators such as uridine and cytidine, factors necessary for CNS tissue recovery were significantly lower in the Ron^−/−^ CNS (Figure 2G) (45). These experiments indicate that homeostatic CNS expression of Ron plays a protective role in regulating neuroinflammation and metabolic homeostasis.

### Ron Is Neuroprotective in EAE

**Figure 3:**
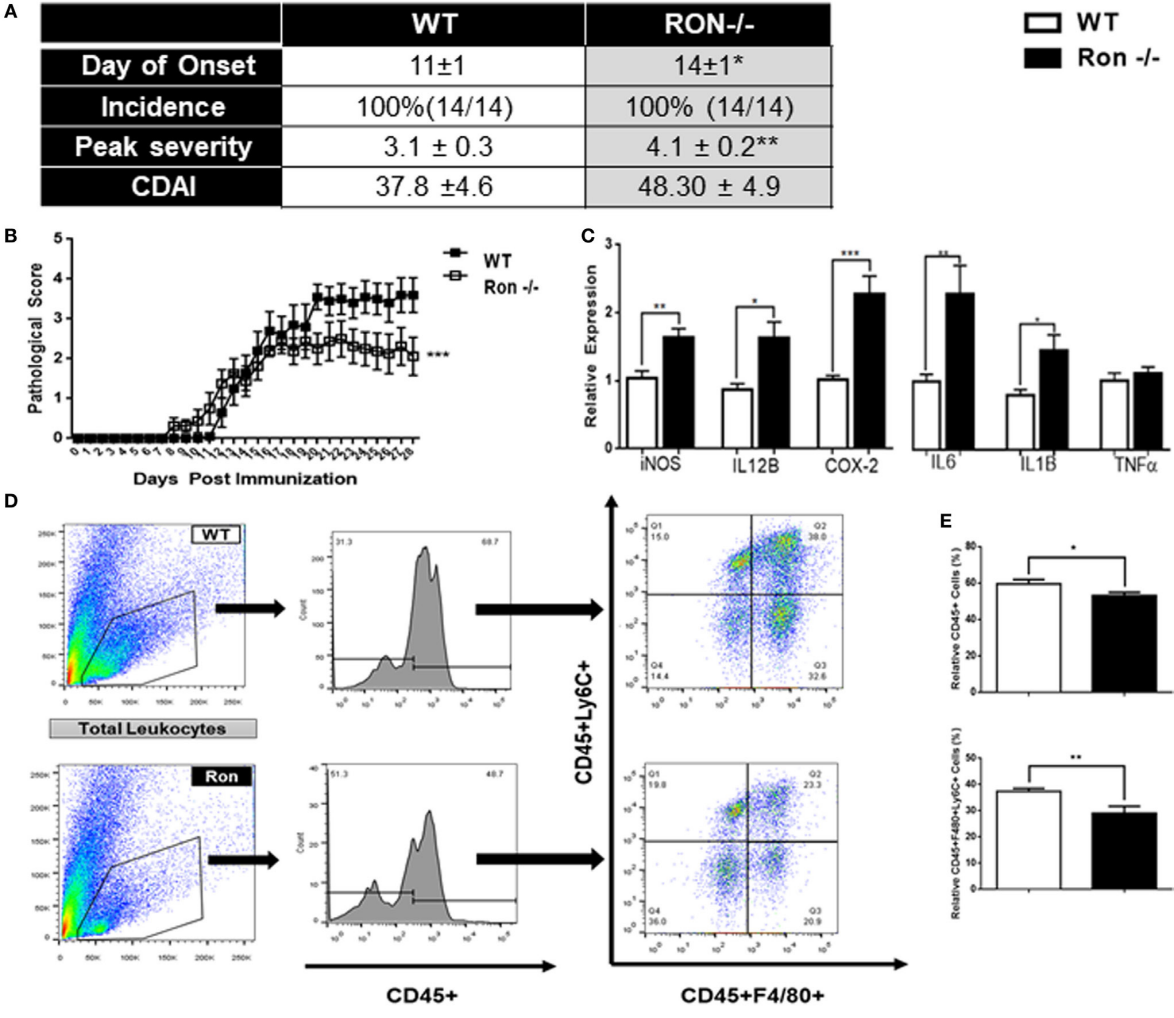

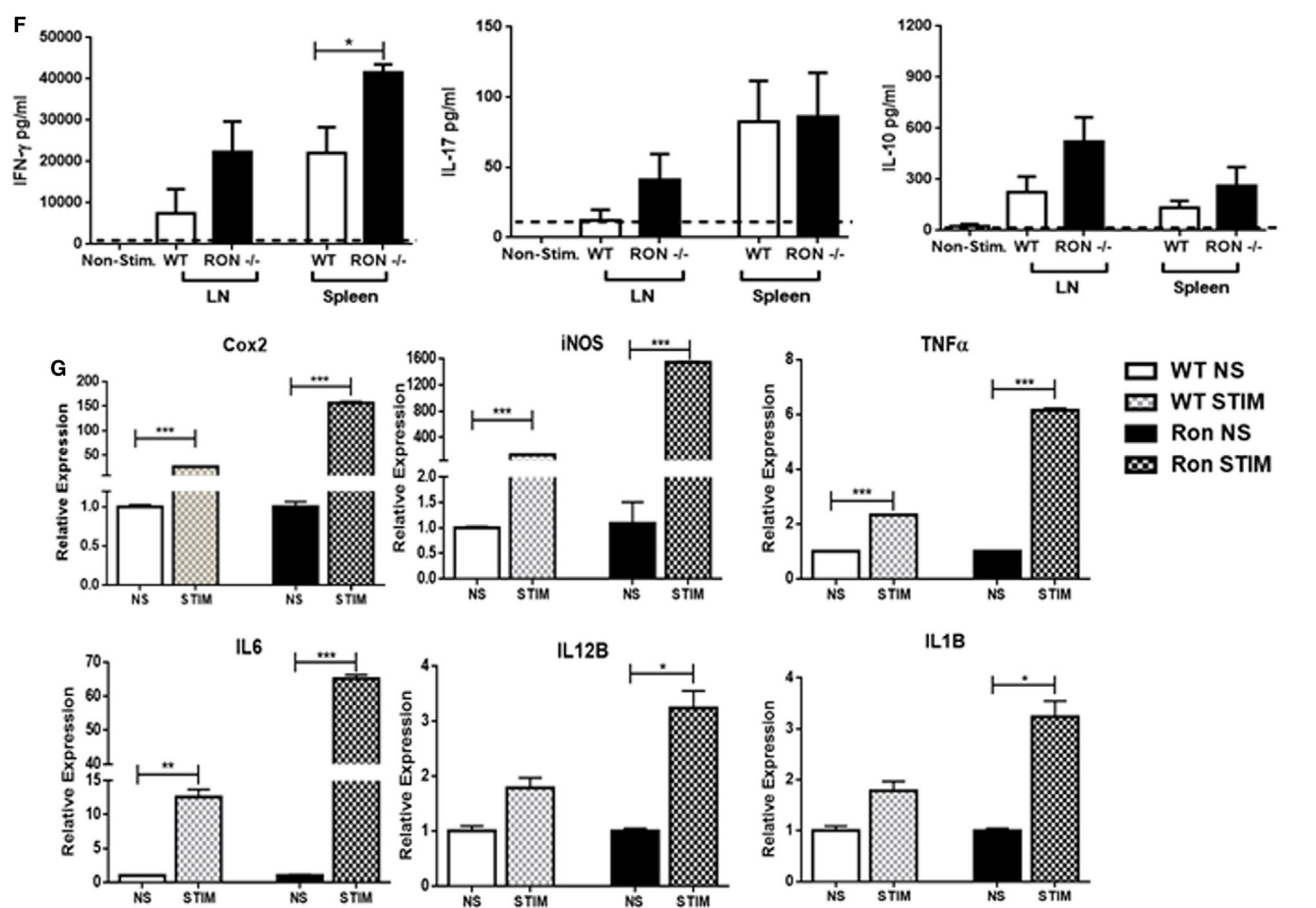
Ron plays a protective role in the progression of experimental autoimmune encephalomyelitis. **(A,B)** Wild-type (WT) and Ron^−/−^ mice were immunized with myelin oligodendrocyte glycoprotein (MOG) at 8–10 weeks of age and disease progression was monitored daily for 27 days (*n* = 14 mice/genotype). **(C)** Expression of pro-inflammatory genes in the central nervous system (CNS) of WT and Ron^−/−^ mice 14 days following immunization with MOG (*n* = 6 mice/genotype). **(D,E)** Total CNS immune cells were isolated from WT and Ron^−/−^ mice 14 days following immunization and the presence of infiltrating CD45^+^F480^+^CD206^+^ cells were assessed by flow cytometry (*n* = 6 mice/genotype). **(F,G)** Inguinal and axillary lymph nodes and spleens were isolated from WT and Ron^−/−^ mice at day 14 following immunization and restimulated with MOG for 72 h. Levels of IFN-γ, IL-17, and IL-10 in the supernatant was determined by ELISA and expression of pro-inflammatory cytokines was assessed by qPCR (*n* = 6 mice/genotype) (*P < 0.05, **P < 0.01, ***P < 0.001).

Although MS is characterized by inflammation largely attributed to an adaptive T cell-mediated autoimmune response, the innate immune system regulates disease onset and progression. In order to determine whether Ron plays a protective role under disease conditions, we utilized a murine model for MS, EAE. The Ron^−/−^ mice had delayed onset of disease; pathological scores at peak disease state (day 14) were significantly higher in Ron^−/−^ mice compared to age-matched WT controls (Figure 3A,B). Expression of iNOS, IL-12B, Cox2, IL-6, and IL-1B, was significantly higher in d14 CNS tissue from Ron^−/−^ mice than in d14 WT mice (Figure 3C). Surprisingly, Ron^−/−^ CNS tissue had decreased percentages of CD45^+^ immune cells and infiltrating Ly6C^+^F480^+^ monocytes/macrophages when compared to WT CNS at peak disease state of day 14 (Figures 3D,E). When evaluated across the entire disease spectrum (day 14 and 28), Ron^−/−^ mice presented decreased absolute percentages of F480^+^ and Ly6C^+^ cells only at peak disease state (Figure S1A in Supplementary Material).

In order to determine the extent of peripheral inflammation, inguinal LNs, and spleens of immunized mice were collected at peak disease state (day 14) and were assessed for cytokine secretion following *in vitro* stimulation with MOG. Cells isolated from the spleen, but not the LN, of Ron^−/−^ mice secreted increased levels of IFNγ, but not IL-17 and IL-10, following MOG stimulation (Figure 3F). *In vitro* stimulation of splenocytes from Ron^−/−^ mice with MOG resulted in increased expression of M1-associated genes when compared to splenocytes from WT mice (Figure 3G). We observed an increase in CD4^+^ T cells in the Ron^−/−^ LN with a concomitant decrease in CD8^+^ T cells (Figure S1B in Supplementary Material). Despite lower number of F4/80^+^ macrophages in the Ron^−/−^ LN (Figure S1B in Supplementary Material), we observed a significant increase in the expression of the M1 macrophage-associated genes iNOS, COX-2, IL-6, IL-12B, IL-1β, and TNF-α in unstimulated and MOG-stimulated cells (Figures S1C,D in Supplementary Material). These results demonstrated loss of Ron supports an M1-mediated inflammatory environment in the CNS and periphery in context of EAE.

### DIO Promotes Macrophage-Mediated CNS Inflammation

**Figure 4:**
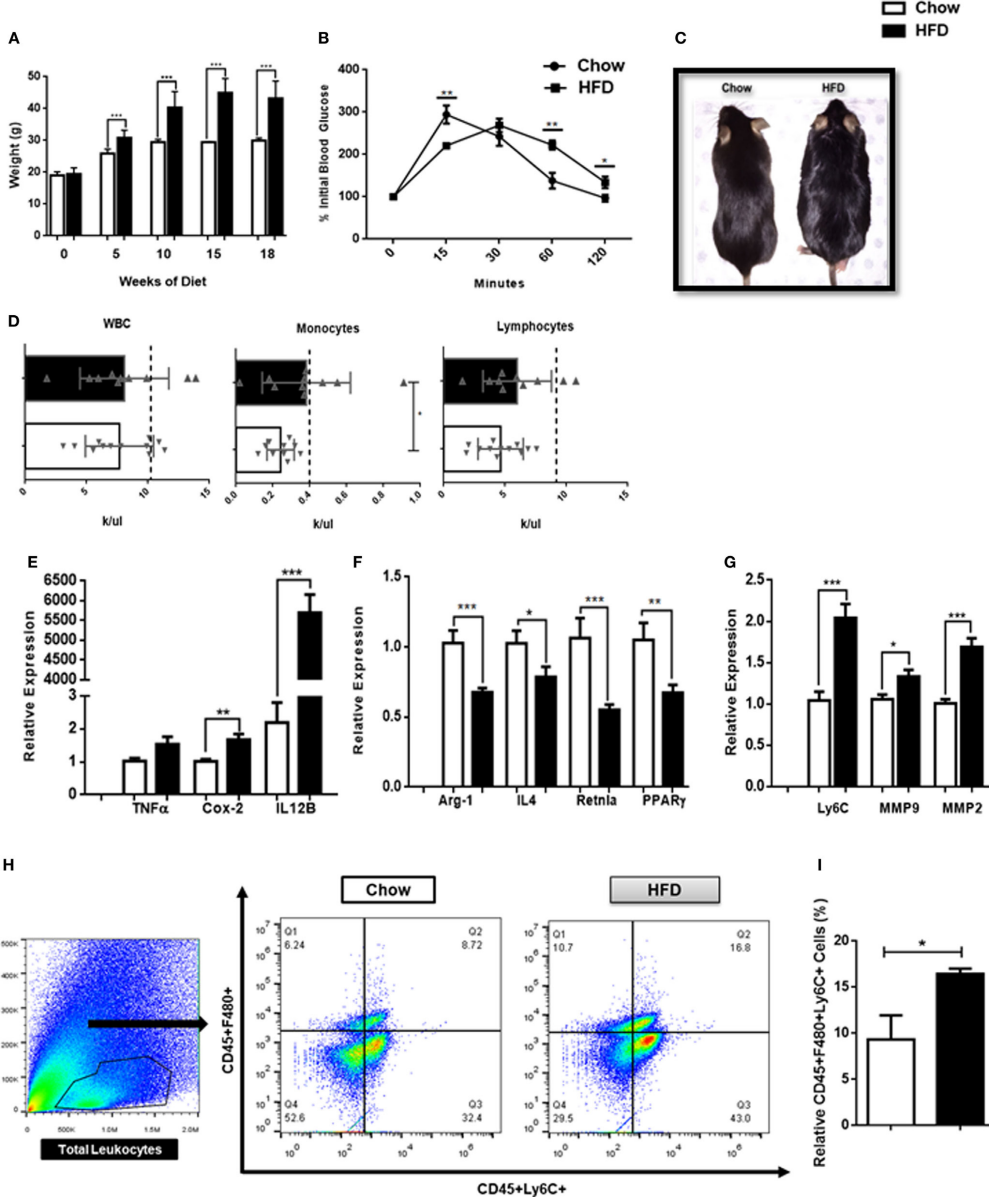
High fat diet-induced obesity promotes inflammation in the central nervous system (CNS). **(A)** Weight of mice following 18 weeks on a HFD or chow controls (*n* = 18 mice/group). **(B)** A terminal intra-peritoneal glucose tolerance test was performed on mice following 18 weeks on a HFD or chow controls (*n* = 6 mice/group). **(C)** Representative body morphology of mice following 18 weeks on a HFD or chow controls. **(D)** Circulating leukocyte counts from mice following 18 weeks on a HFD or chow controls (*n* = 10–12 mice/group). **(E–G)** Mice were maintained on a HFD or chow for 18 weeks and expression of M1-associated genes, M2-associated genes and matrix metalloproteinases (MMPs) in the CNS was assessed by qPCR (*n* = 12 mice/group). **(H,I)** Total CNS immune cells were isolated from mice maintained on a HFD or chow for 18 weeks and the number of CD45^+^F4/80^+^Ly6C^+^ infiltrating monocyte/macrophages was assessed by flow cytometry (*n* = 6 mice/group) (*P < 0.05, **P < 0.01, ***P < 0.001).

It is becoming increasingly clear that obesity is a chronic inflammatory disease. In order to determine whether mice exhibit inflammation in the CNS in the context of a DIO model, 6-week-old WT mice were placed on a HFD for 18 weeks and CNS tissue and blood were collected. Consistent with previous studies, mice maintained on a HFD identified with a significant weight gain throughout the 18-week period when compared with mice maintained on regular chow (Figures 4A,C). As expected, mice on the HFD also had impaired blood glucose tolerance at 18 weeks of age when compared with mice fed normal chow (Figures 4B,C). Both male and female mice experienced weight gain following 18 weeks on a HFD (Figure S2 in Supplementary Material). Mice fed a HFD had significant increases in circulating monocytes but not lymphocytes or total circulating WBCs (Figure 4D). Interestingly, CNS tissues from these animals identified with elevated expression of COX-2 and IL-12B genes, associated with M1-mediated inflammation (Figure 4E) and decreased expression of the M2-associated genes, Arg1, IL-4, Retnla, and PPARγ (Figure 4F). Furthermore, CNS tissue from mice maintained on a HFD also had increased expression of Ly6C, MMP2, and MMP9, indicative of focal inflammation, matrix degradation, and potential BBB breakdown at the parenchymal borders (Figure 4G). There was also increased trafficking of monocytes (F480^+^Ly6C^+^) into the CNS proper following 18 weeks on a HFD (Figures 4H,I). Consistent with previous studies, these results demonstrate that there is an altered balance in macrophage heterogeneity associated with DIO-associated neuroinflammation.

### Loss of Ron Exacerbates Neuroinflammation in DIO

**Figure 5:**
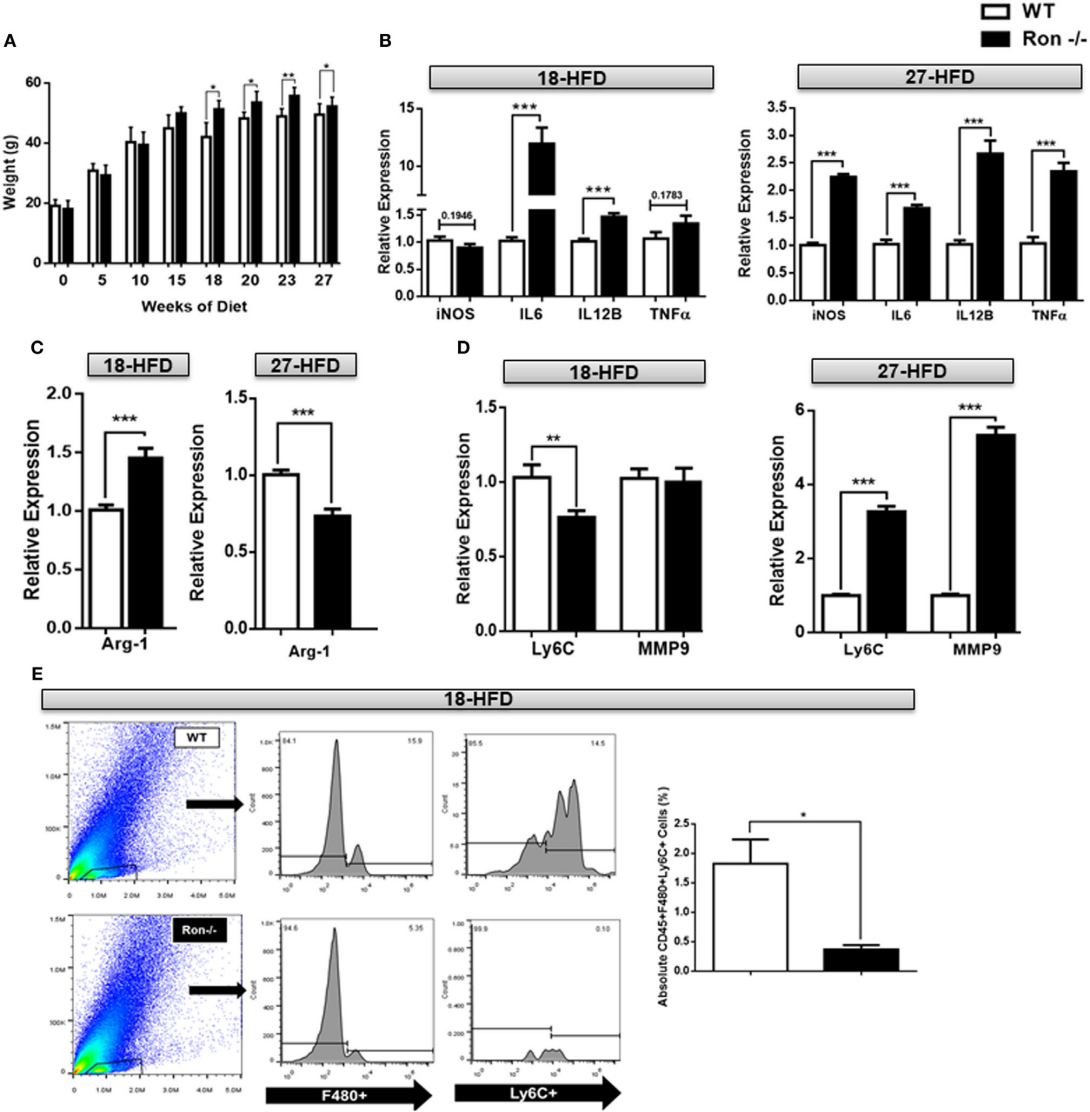

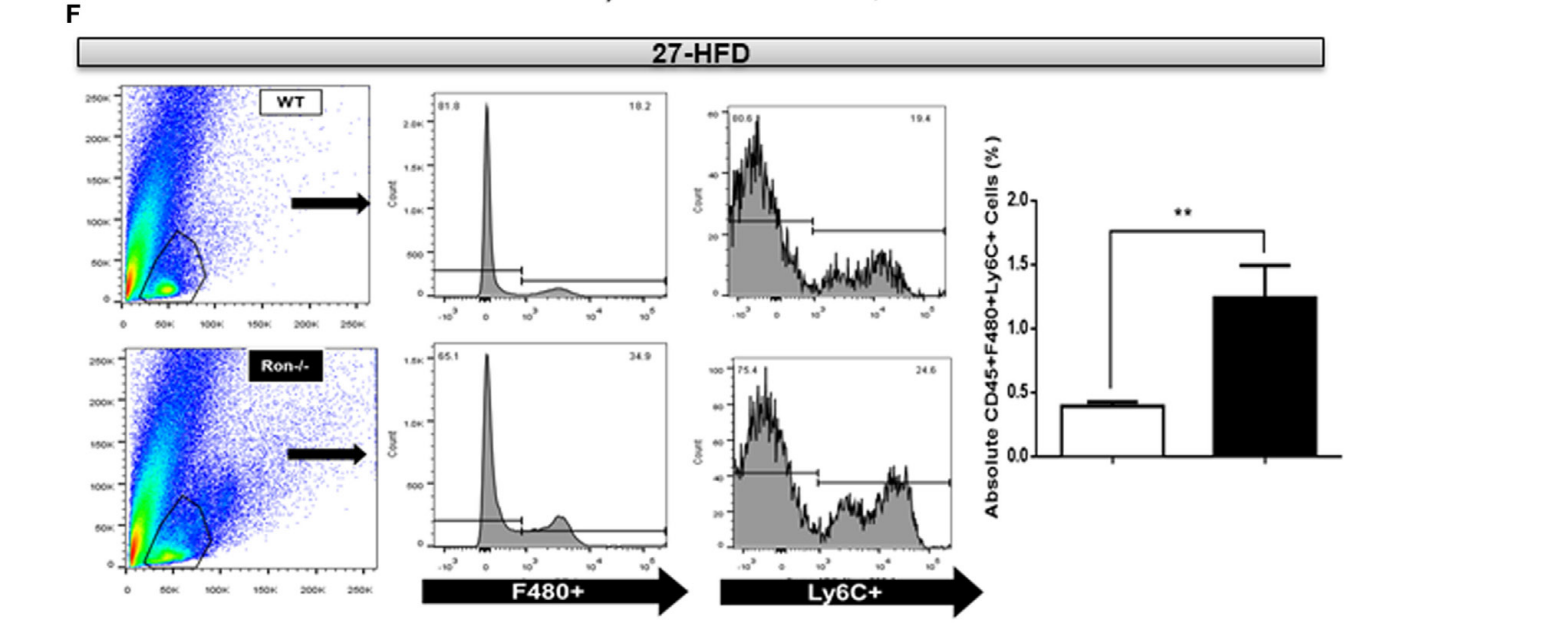
Loss of Ron stimulates exacerbated neuroinflammation following diet-induced obesity. **(A)** Weight of wild-type (WT) and Ron^−/−^ mice maintained on a HFD for 18 (*n* = 16 mice/genotype) and/or 27 weeks (*n* = 16 mice/genotype). **(B–D)** WT and Ron^−/−^ mice were maintained on a HFD for 18 or 27 weeks and expression of M1 genes, M2-gene Arg-1, Ly6C, and matrix metalloproteinases (MMPs) in the central nervous system (CNS) was assessed by qPCR (*n* = 6 mice/genotype). **(E,F)** Total CNS immune cells were isolated from the CNS of WT and Ron^−/−^ mice maintained on a HFD for 18 or 27 weeks and numbers of CD45^+^F480^+^Ly6C^+^ infiltrating macrophages was assessed by flow cytometry (*n* = 8 mice/genotype) (*P < 0.05, **P < 0.01, ***P < 0.001).

We have previously shown that Ron regulates macrophage heterogeneity both *in vitro* and *in vivo*, particularly in tissue inflammation associated with diet-induced hepatosteatosis and atherosclerosis (27). To determine whether a loss of Ron influences chronic neuroinflammation, 6-week-old WT and Ron^−/−^ mice were placed on a HFD diet for 18 and/or 27 weeks. Ron^−/−^ mice gained more weight when compared to their WT counterparts, as early as 15 weeks, which was maintained through 27 weeks (Figure 5A). At 18 weeks of HFD CNS tissue from Ron^−/−^ mice sustained increased expression of the inflammatory genes, IL-6 and IL-12B (Figure 5B) and also increased expression of M2-associated genes including Arg1, Ym-1, and VegF (Figure 5C). However, CNS tissue from Ron^−/−^ mice highlighted decreased expression of Ly6C and MMP2 at this time point, gene-associated CNS degradation or trafficking of monocytes into the CNS proper (Figure 5D,E). Consistently, flow cytometry revealed decreased infiltration of F480^+^/Ly6C^+^ monocytic macrophages in the CNS of Ron^−/−^ mice. Following 27 weeks of HFD CNS tissue exhibited exacerbated expression of the iNOS, IL-6 IL-12B, and TNFα, genes associated with M1-mediated inflammation (Figure 5B), and decreased expression of the M2-associated gene, Arg1 (Figure 5C). At this timepoint, brains from Ron^−/−^ mice maintained on a HFD also displayed increased expression of Ly6C, and MMP9 compared to diet-matched WT animals, indicative of severe focal inflammation and loss in BBB integrity (Figure 5D). Also at 27 weeks, Ron^−/−^ mice displayed elevated monocyte trafficking into the CNS associated with increased Ly6C^+^ cells (Figure 5F). This result indicates the potential role of chronic stressors such as DIO in neurodegeneration and highlights the potential protective role of Ron in long-term neuroinflammation associated with obesity.

## Discussion

Tissue-resident macrophages play a critical role in regulating local inflammation in the periphery, and microglia are no different in the context of neuroinflammation. Macrophage heterogeneity governs CNS homeostasis whereby quiescent M2-like microglia maintain housekeeping functions and repair, while a loss of M2 macrophages has been implicated in age-mediated neurodegenerative diseases such as AD (20). Though the sleeping brain engages in pro-inflammatory activity for debris and residual plaque clearance mediated through M1-activation, the grade of neuroinflammation in that case is closely regulated through a balance between M2 and M1 macrophages (46, 47). This inhibition checkpoint mediated by M2 macrophages attenuates M1-mediated tissue injury and uncontrolled inflammation. Our lab and others have demonstrated that treatment of macrophages with the Ron agonist MSP *in vitro* results in the inhibition of M1 macrophage activation in favor of the M2 phenotype (29–32, 48). By contrast, animals with a targeted deletion of the MSP binding site in Ron exhibit elevated levels of systemic and circulating IFNγ as well as IL12p40 following induction of septic shock (48). In experimental models of MS, DSS induced colitis, and lung injury, Ron plays a protective role through attenuation of TNFα in the periphery and CNS (49, 50). A loss of Ron exacerbates obesity and the development of atherosclerosis mediated and impaired glucose tolerance, associated with increased hepatic lipid accumulation and greater aortic plaque burden (27). These studies highlight the protective role of Ron in regulating CNS tissue and cellular inflammation in the context of DIO.

Consistent with results from resident and peritoneal macrophages, MSP treatment significantly inhibited the expression of TNFα and IL-1B in these cells following LPS stimulation, confirming the capacity of MSP to similarly alter the inflammatory phenotype of microglia.

An intriguing finding was the role of Ron in tissue homeostasis in the CNS, whereby a loss of Ron induced neuroinflammation without any particular stress stimulus. This baseline level of CNS inflammation was associated with a decrease in genes associated with an M2 macrophage phenotype and a concomitant increase in M1-associated neuroinflammation. The metabolite analysis indicated that a loss of Ron served as a potent stressor to the CNS metabolic landscape. Metabolites such a choline and glutamate are indicators of significant neurotoxicity. Glutamate has more recently been identified as a target of the NFκB pathway, and NFκB activation correlates directly with increased CNS glutamate levels in neuropathological cases. Consistent with the increased glutamate, previous studies in our lab has shown that Ron inhibits inflammatory M1 macrophage activation by limiting TRAF6-activation of I kappa B kinase and subsequent NFκB activation mediated by LPS stimulation of TLR4 in primary macrophages *in vitro*.

Our studies confirmed previous results that introduced the protective role of Ron in EAE (50). In addition, here we demonstrated that increased pathological severity was coincident to increased M1-mediated macrophage inflammation in the CNS proper. The Ron^−/−^ mice also displayed a largely adaptive inflammatory response in the periphery, as demonstrated by the significant increase in splenic IFNγ production, but not IL-17 and IL-10. The results from this study suggest a crosstalk between the innate and adaptive immune system, which may be in part influenced by the activation of Ron in the periphery.

Longitudinal studies in humans have solidified the stimulatory role of obesity in neurodegenerative cases such as earlier onset of AD. Through these studies, we demonstrated the potential importance of tissue macrophage heterogeneity in the underlying neuroinflammatory landscape. Evaluating the role of Ron in chronic inflammation further confirmed the protective role of Ron in the progressive stages of uncontrolled inflammation. In a DIO model, the absence of Ron initially resulted in an elevation of both M1- and M2-associated genes; however with chronic exposure to DIO, the loss of Ron signaling promotes enhanced neuroinflammation with a classically activated M1 phenotype eventually counterbalancing the M2 phenotype. Early or acute inflammation triggers both M1 and M2 macrophage phenotypes in the CNS as noted previously in murine models of SCI, in which case the CNS attempts to engage in repair processes. Persistent or chronic loss of Ron, perhaps initiates disinhibition of the M1 cascade and supports uncontrolled inflammation as observed in later stages of DIO.

Ron is widely expressed in the CNS in both microglia and neurons. Ongoing and future studies are focusing on the cell specific role underlying the protective role of Ron expression in neuroinflammation. Understanding the significance of Ron in neuronal cells may shed further light on the cellular mechanisms by which Ron is protective during CNS development and in progressive disease states. Therapeutic strategies that engage in the switch from a pro-inflammatory phenotype to a quiescent M2-like phenotype or the activation of Ron in the CNS may have the potential to alleviate the uncontrolled progression of inflammation underlying various neurodegenerative diseases.

## Ethics Statement

This study was carried out in accordance with the recommendations of the Pennsylvania State University Institutional Animal Care and Use Committee. The protocols were approved by the Pennsylvania State University Institutional Animal Care Committee.

## Author Contributions

AD conceived and carried out the experiments, analyzed the data, and helped to write the manuscript. JA helped design and perform the experiments. JF helped perform the experiments and write the manuscript. LS helped perform the EAE experiments. YT helped perform and analyze the metabolomics experiments. LZ helped perform and analyze the metabolomics experiments. RP helped design the experiments and provided critical reagents. AP provided expertise in metabolomics and help designing the experiments. MC provided expertise in EAE and help designing the experiments. PH-G helped conceive and design the experiments, analyze the data, write the manuscript, and oversaw the project.

## Conflict of Interest Statement

The authors declare that the research was conducted in the absence of any commercial or financial relationships that could be construed as a potential conflict of interest.
