# Supplementary material for: Neuroprotective Role of the Ron Receptor Tyrosine Kinase Underlying Central Nervous System Inflammation in Health and Disease
Source: Front Immunol. 2018 Mar 19;9:513. doi: 10.3389/fimmu.2018.00513 (PMC5868034; doi:10.3389/fimmu.2018.00513)
Supplement: Supplementary file 1 [file Image_1.PDF]

Supplementary Material

Neuroprotective Role of the Ron Receptor Tyrosine Kinase underlying CNS Inflammation in Health and Disease

Adwitia Dey,[1] Joselyn N.Allen,[1] James W.Fraser,[1] Lindsay Snyder,[1] Yuan Tian,[1] Limin Zhang,[1] Robert F. Paulson,[1] Andrew Patterson,[1] Margherita T. Cantorna,[1] and Pamela Hankey-Giblin*[1]

*** Correspondence:** Dr.Pamela Hankey-Giblin phc7@psu.edu

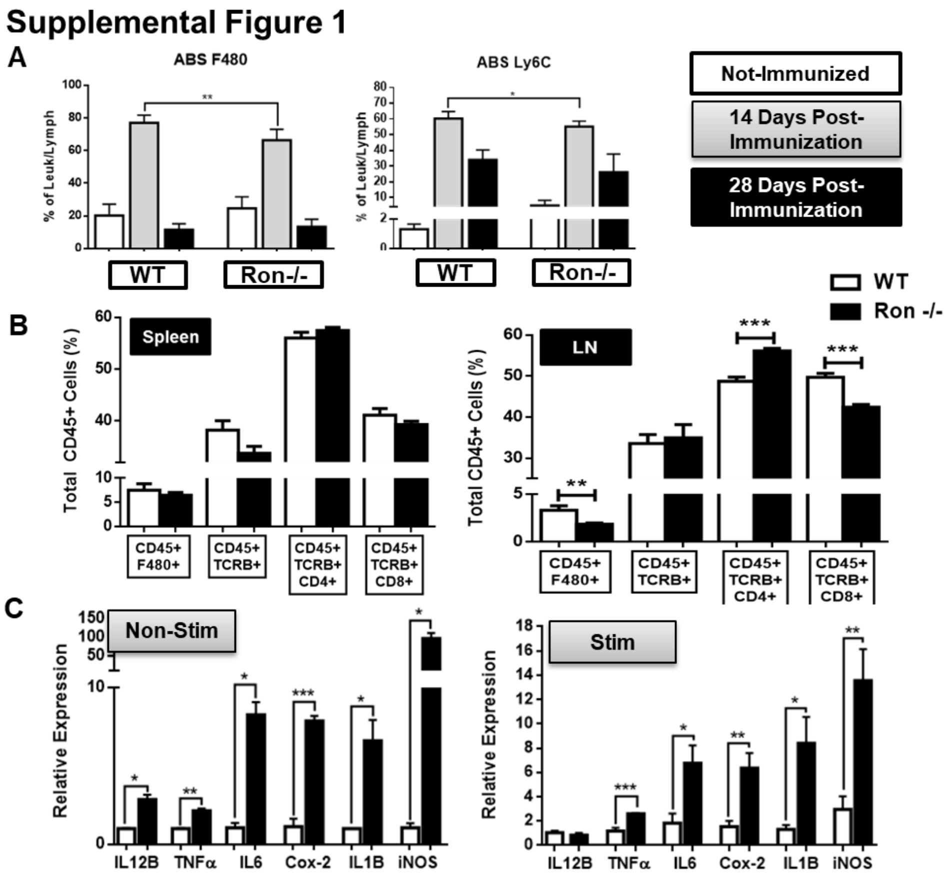

Supplementary Figure 1. Loss of Ron supports an M1-mediated inflammatory environment in the CNS and periphery in peak disease state of EAE

(A) Total CNS immune cells were stained for absolute total macrophage (F480+) and trafficked macrophage (Ly6C+) population non-immunized, at 14 and 28 days following immunization with MOG (n=6/genotype/condition). **(B)** Total immune cells were isolated from spleens and inguinal lymph node (LN) and characterized for macrophages (CD45+F480+) or T-cell populations (CD45+TCRB+CD4+/CD8+) 14 days following immunization with MOG (n=8/genotype/condition). **(C)** LN cell populations were isolated and re-stimulated with MOG *in vitro* and assessed for expression of pro-inflammatory genes 14 days following immunization with MOG (n=6/genotype). *P<0.05, **P<0.01, ***P<0.001.

Supplemental Figure 2

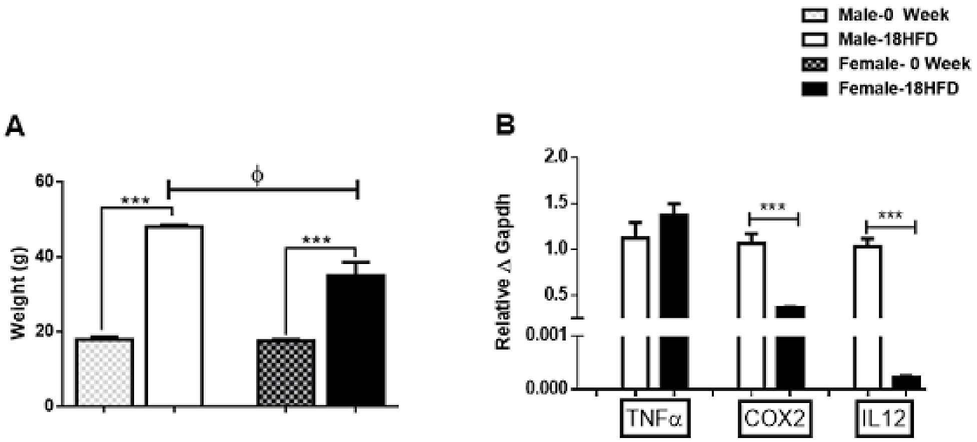

Supplementary Figure.2 Diet-induced obesity associated CNS inflammation is exacerbated in male compared to female WT mice

(A) Male and Female age matched C57BL6 were placed on a high fat diet(HFD) 18 weeks and weighed (grams) at weeks 0 and following 18 weeks of HFD (N=6/sex/condition). **(B)** Expression of pro-inflammatory genes TNFα, COX-2, IL-12B in the HFD CNS of both males and females was assessed by qpcr. (n=6 mice/sex) *P<0.05, **P<0.01, ***P<0.001.
